# Comparison of genetically modified insect-resistant maize and non-transgenic maize revealed changes in soil metabolomes but not in rhizosphere bacterial community

**DOI:** 10.1080/21645698.2022.2025725

**Published:** 2022-02-18

**Authors:** Yanjun Chen, Libo Pan, Mengyun Ren, Junsheng Li, Xiao Guan, Jun Tao

**Affiliations:** a College of Tropical Crops, Hainan University, Haikou, P.R. China; b State Key Laboratory of Environmental Criteria and Risk Assessment, Chinese Research Academy of Environmental Sciences, Beijing, P.R. China; c Institute of Crops and Nuclear Technology Utilization, Zhejiang Academy of Agricultural Sciences, Hangzhou, P.R. China

**Keywords:** Crop biotechnology, bt crops, microbial community, root exudates, environmental safety, sustainable agriculture

## Abstract

The deliberate introduction of the beneficial gene in crop plants through transgenic technology can provide enormous agricultural and economic benefits. However, the impact of commercialization of these crops on the ecosystem particularly on belowground soil biodiversity is still uncertain. Here, we examined and compared the effects of a non-transgenic maize cultivar and an insect-resistant transgenic maize cultivar genetically engineered with *cry1Ah* gene from *Bacillus thuringiensis*, on the rhizosphere bacterial community using 16S rDNA amplicon sequencing and soil metabolome profile using UPLC/MS analysis at six different growth stages. We found no significant differences in bacterial community composition and diversity at all growth stages between the two cultivars. The analysis of bacterial beta-diversity showed an evident difference in community structure attributed to plant different growth stages but not to the plant type. In contrast, the soil metabolic profile of transgenic maize differed from that of the non-transgenic plant at some growth stages, and most of the altered metabolites were usually related to the metabolism but not to the plant-microbe interaction related pathways. These results suggest that genetic modification with the *cry1Ah* gene-altered maize soil metabolism but had no obvious effect on the rhizosphere bacterial community.

## Introduction

With the ever-growing human population, food security is the major concern of this century.^1^ Thus, the agriculture sector has been revolutionized to obtain higher crop yield per capita through various sustainable approaches. Genetically modified (GM) crops, as a result of the introduction of beneficial genes in a crop plant, can provide sustainable agronomic and economic benefits.^2^ For instance, when a gene from *Bacillus thuringiensis* (Bt) responsible for the production of insecticidal toxin is introduced into certain crops, it allows protection of GM plants against a specific group of insect pests through the direct action of insecticidal toxin.^3^ Since their first commercialization in 1996, several GM crops such as insect-resistant, herbicide-resistant, combined insect- and herbicide-resistant and viral disease-resistant crops are being cultivated in different parts of the world.^4,5^ Although the global cultivable area of GM is increasing every year, their effects on ecosystem biodiversity remain controversial.

Some serious agricultural concerns associated with the commercial cultivation of GM crops have been reported so far.^6^ In Bt crops, for example, the target insect pests may develop Bt toxin resistance over time^7,8^ (Guan, Hou, Dai, Liu, Liu, Gu, Jin, Yang, Fabrick, Wu, 2021). Another concern is the transgene flow from Bt crops to surrounding plant diversity, and the potential development of ‘super weed’ is one of its examples.^9^ Similar to aboveground, the study of consequences of commercial cultivation of Bt crops on belowground biodiversity is of equal importance, as the health and performance of crop plants is highly dependent on soil biological processes.

Soil microbial community is a principal component of soil ecosystem functioning.^10,11,12^ Plant root harbors a mesmerizing diversity of microbes mainly dominated by the bacterial community in their ambient environment that is called the rhizosphere.^13,14^ These rhizosphere bacteria, ranging from plant pathogenic to beneficial ones, have great influences on host plant health as some may regulate plant nutrient acquisition ability or modulate host immunity.^15–17^ Plant actively secretes photosynthetically fixed carbon in the shape of root exudates, usually consisting of primary and secondary metabolites, into the soil, which acts as the energy source for the rhizosphere microbial community^18^ (Rahman et al.,^19^ 2021). This phenomenon gives hosts to preferentially select and shape their rhizosphere microbiota through modification in root exudation.^20–23^

GM plants also secrete these metabolites to determine root-associated microbiota. The expression of specific proteins from GM plants may alter the composition of root metabolites, thereby the composition of root-associated microbiota, which may, in turn, affect the soil biological environment.^24^ So far, contrasting effects of GM crops on rhizosphere microbial community have been reported with a plethora of studies suggesting no obvious effect,^25–28^ while some studies showing significant changes in the rhizosphere microbial community as compared to non-GM crops^29^ (Guan, Wei, Stewart, Tang, 2021). Studies also indicate that other factors such as the plant growth stage could be a major indicator of changes in the rhizosphere microbial community.^30,31^ These studies suggest the necessity of assessing the effects of a given genetically modified crop on its rhizosphere microbial community before its commercialization.

The Cry1Ah protein of *cry1Ah*, a novel insecticidal gene from Bt subspecies, exhibits great toxicity to Lepidopteran insects, and its efficiency has been found higher than other insecticidal genes such as *cry1Ab* and *cry1Ac*.^32,33^ The expression of Cry1Ah might lead to altering plant root metabolites composition to induce changes in ambient soil biodiversity.^24^ Although we found no significant effects on weeds, nematodes and other invertebrate occurrences in *cry1Ah* genetically engineered maize HGK60 surroundings as compared to its non-transgenic control,^34,35^ its effects on the rhizosphere microbial community remain to be explored. Here, we used 16s rDNA amplicon sequencing to study the changes in rhizosphere bacterial community composition, and non-targeted metabolomics for soil metabolite profiling of transgenic and non-transgenic maize.

## Materials and Methods

### Plant Material and Field Experiment

The seeds of GM maize HGK60 with insecticidal gene *cry1Ah* and non-transgenic maize ZHENG58 (control) were provided by the Chinese Academy of Agricultural Sciences, China. The field experiment was carried out from May to September 2020 in an open field at the experimental station (116°36’34’’E, 39°36’10’’N) of the Chinese Academy of Agricultural Sciences located in Langfang, China, with a tropical monsoon climate, an average lowest-highest temperature of 24.3–28.8°C, and average monthly precipitation about 122 mm for the plant growth period. The experimental site has been cultivated with HGK60 maize for more than 10 years.

Both maize types were planted in separate plots (10 m × 10 m) in a randomized block design with six replicates for each type (~12 plots). Plots were separated from each other with a 1 m wide uncultivated zone. The sowing of seeds in each plot was carried out with inter-plant spacing of 25 cm and inter-row spacing of 60 cm. The plots were managed conventionally.

### Sample Collection

Rhizosphere soil samples from both maize types were collected at six different growth stages, i.e., pre-planting stage (April 28), seedling stage (May 10), bell stage (June 10), heading stage (June 30), fully ripe stage (August 10), and after-harvest stage (September 10). Briefly, the maize plant roots were carefully removed from the soil and shaken by hand to remove loosely attached soil (not rhizosphere soil). Then, soils tightly adhering to roots were removed by a sterile brush (rhizosphere soils). To limit the influence of soil heterogeneity, the preparation of composite soil samples for DNA extraction is recommended (Vestergaard et al.,^36^ 2017). Therefore, a composite sample was prepared from random ten maize plants in each replicate of the individual treatment. This resulted in six composite rhizosphere soil samples for each treatment. After sieving (2 mm mesh), these fresh rhizosphere soil samples were stored at −80°C for DNA extraction.

### DNA Extraction, Illumina MiSeq Sequencing and Raw Data Processing

Soil DNA was extracted from 0.5 g of rhizosphere soil using a FastDNA ® SPIN Kit for soil (MP Biomedicals, Santa Ana, CA) following the manufacturer’s instructions. The concentration and purity of DNA was confirmed by an ultra-micro spectrophotometer.

The V1-V9 region of the bacterial gene was amplified in PCR assay using the universal primer set of 8 F (5′-AGAGTTTGATCCTGGCTCAG-3′) and 1509 R (5′-GNTACCTTGTTACGACTT-3′). The PCR reaction (50 µL) was: Trans Fastpfu 1 µL, 5 x Buffer 10 µL, 5 x Stimulate 5 µL, dNTPs (2.5 × 10^−3^ mol/L each) 5 µL, Primer Mix (1 µmol/L) 2 µL, gDNA 1 µL, NFW 26 µL. The conditions for PCR reactions were: Pre-denaturation temperature 98°C (2 min), denaturation temperature 95°C (30 s), annealing temperature 60°C (45 s), extension temperature 72°C (90 s), after 35 cycles, the extension was terminated at 72°C for 10 min. The product of the triplicate reaction was pooled and purified using an Agarose Gel DNA purification kit (TaKaRa). Then, a TBA-380 micro-fluorometer with PicoGreen reagent was used to quantify the purified amplicons. The third generation full-length 16S amplicon sequencing was then performed on PacBio platform at Novogene Co. Ltd., Beijing, China.

The obtained raw reads were demultiplexed, quality filtered and further processed using FLASH.^37^ Operational taxonomic units (OTUs) were generated by binning the unique sequences at 97% sequence similarity with the help of an agglomerative clustering algorithm using UPRASE.^38^ The classification of sequences of OTUs was carried out with SILVA database.^39^ The identification and removal of chimeric sequences were done with the help of UPRASE 6.1 in QIIME.^40^ The data of all the sequences was uploaded in NCBI Sequence Archive with the submission accession number (PRJNA75527).

### Metabolites Extraction from Maize Soil, Untargeted Metabolomics Analysis and Raw Data Processing

The procedure described by,^41^ was used for the collection of maize soil metabolites with some modifications. Briefly, maize plants with whole root system were collected and made sure that most of the root-soil was retained, and placed in a pot (size varied with the plant age). For pre- and post-harvest stages, only soil (100 g) for each sample was collected and used. 50% methanol solution (0.05% formic acid, v/v) was applied (15 mL for pre-harvest, seedling and post-harvest growth stage for 1 min each, and 30 mL for the remaining three growth stages for 2 min each) and flushed through the pot with pressure using a syringe. After that, 10 mL of extract was collected in a centrifuge tube, and centrifuged to pellet soil residues (5 min, 3500 *g*). The supernatant (4 mL) was collected and transferred to a new centrifuge tube and initially frozen in liquid nitrogen. Then, the samples were freeze-dried for 48 hours and stored at −80°C for metabolites analysis.

A total of 72 samples (2 treatments × 6 growth stages × 6 replicates) were re-suspended in a 100 µL of solution (50% methanol, 49.9% water, 0.1% formic acid; v/v), sonicated at 4°C, and centrifuged at 14000 *g* for 15 min at 4°C. The supernatant (80 µL) was then transferred into glass vials containing a glass insert before the analysis on a UPLC system (Thermo, Ultimate 3000LC). Hyper gold C18 (1.9 μm internal diameter) column eluted with a multistep gradient throughout 0.3 mL/min at 40°C was used. The gradient used was consisted of A (94.9% water, 5% acetonitrile, and 0.1% formic acid), and B (99.9% acetonitrile, and 0.1% formic acid). The UPLC system was coupled with a mass spectrometer (Triple-TOF 5600) coupled with an electrospray ionization source. Data acquisition was carried out in full scan mode along with IDA mode. The analysis conditions set for mass spectrometry were as follows: Ion source temperature, 550°C (for both + and – ion); ion spray voltage 5500 V (+) and 4500 V (-); collision energy 10 ev (for both + and – ion) interface heater temperature 550°C (+) and 600°C (-), and curtain gas 35 PSI. The QCs were injected at regular time intervals throughout the run to provide data that can be assessed repeatedly.

The raw data acquired from UPLC-MS were processed with the help of progenesis QI software (Waters Corp., USA). The threshold values set were as: precursor tolerance, 5 ppm; fragment tolerance, 10 ppm; and retention time, 0.02 min.^42^ The data were obtained with m/z, peak retention time and peak intensities. The retention time m/z was used as the ion identifier. Any peak with a missing value was removed. The targeted Peak Finding function in Master View 1.0 software was used to match the molecular formula with the published known compounds in the database. For non-targeted peak findings: we imported the data into the Marker View 1.2.1 software, matched and picked the chromatographic peak using peak finding options. The taxonomic identification of metabolites was carried out online on The Human Metabolome Database and LIPID MAPS using the matched formula. Data from both negative and positive ions were combined for bioinformatics analysis in R (version 4.1.1). Finally, the KEGG pathways were identified and constructed at KEGG online (http://www.genome.jp/kegg/).

### Statistical Analysis

The alpha diversity indices of the rhizosphere bacterial community including species richness and Shannon index were calculated using QIIME.^40^ The bacterial beta diversity analysis, the weighted UniFrac distances, were calculated using QIIME. For estimation of dissimilarity in bacterial community structure, principal component analysis (PCA), principal coordinate analysis (PCoA) and Bray-Curtis dissimilarity matrix calculations were conducted in the *vegan* package and visualized in *ggplot2* package in R.

The data of alpha diversity and bacterial differential relative abundance were analyzed by analysis of variance (ANOVA), and means were compared based on student t-test. False discovery rate (FDR) was calculated for bacterial and soil metabolites differential analysis using *edgeR* package in R. The significance of data based on FDR value (<0.05) was considered with a fold change threshold (log_2_foldchange >1 or <-1). The differences in soil metabolites between two maize cultivars were compared using partial least square discriminant analysis (PLS-DA). Graphs were visualized using the *ggplot2* package in R.

## Results

### Alpha and Beta Diversity of Maize Rhizosphere Bacterial Community

**Figure 1.**
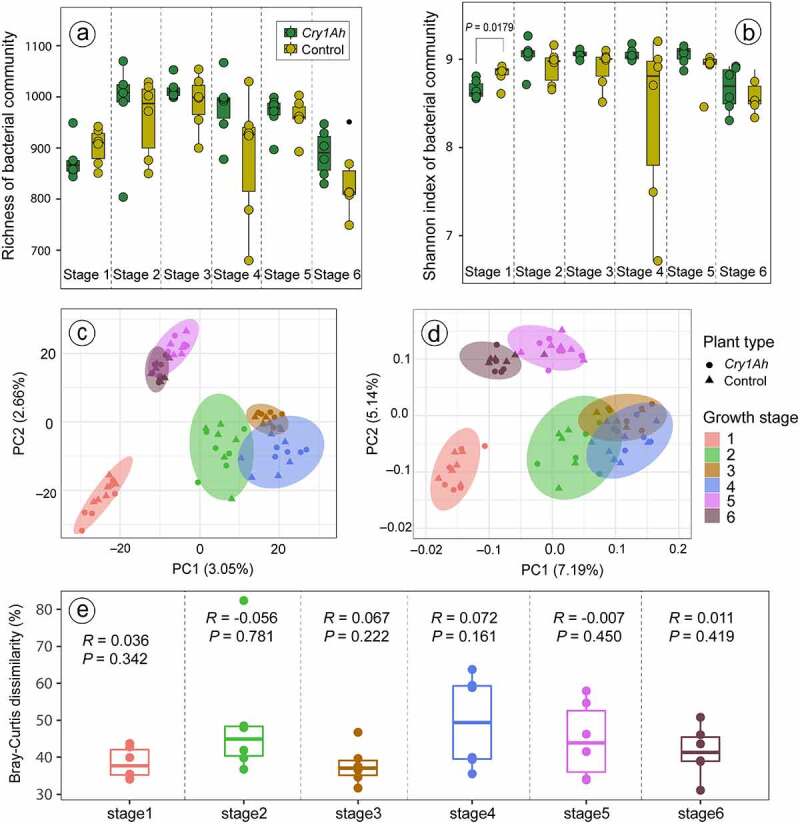
The species richness (a), Shannon diversity index (b), beta diversity calculated via principal component analysis (c) and principal coordinate analysis (d) based on UniFrac distance of the bacterial community, and Bray-Curtis dissimilarity (e) in the rhizosphere of insecticidal transgenic and control maize cultivars at six different growth stages. The *P*-value depicts the significant difference in diversity parameter based on the student’s t-test (*P* < .05).

The transgenic maize did not affect the rhizosphere bacterial alpha diversity as the values of alpha diversity indices were not different between transgenic and control maize, except the Shannon index which was higher in control maize at the first stage (P < .05) (Fig. 1a, b). PCA and PCoA analysis based on UniFrac distance showed that the bacterial community distinctly differed among all the six growth stages in both maize cultivars; however, no difference was observed between transgenic and control maize when compared at each stage (Fig. 1c, d). On average, the explained Bray-Curtis dissimilarity ranged from 37.80% to 49.48% between samples of transgenic and control at all six stages (Fig. 1e). However, the analysis of similarities (ANOSIM) revealed that the dissimilarity was not strong (*R*-value close to zero) and statistically non-significant (*P* > .05) at all six stages (Fig. 1e). The results of adonis analysis of comparison of two groups at each growth stage are provided in supplementary file 1.

### Composition of Maize Rhizosphere Bacterial Community

**Figure 2.**
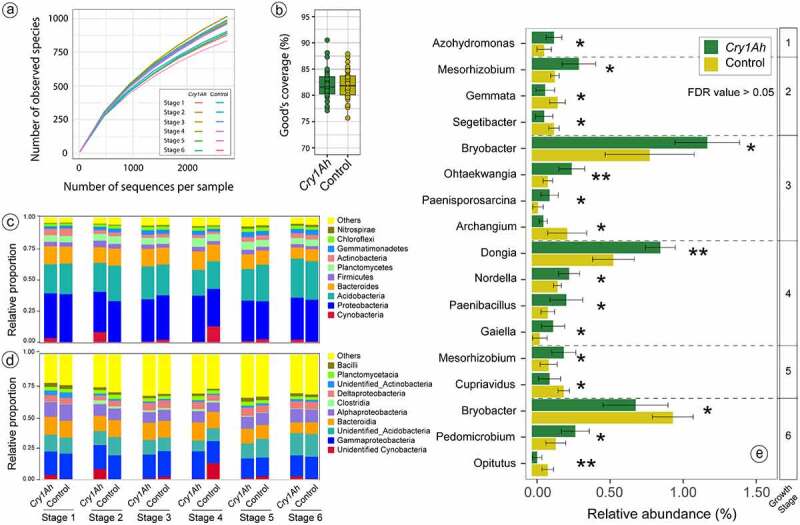
The composition of the rhizosphere bacterial community of insecticidal transgenic and control maize cultivars is estimated by amplicon sequencing. (a) Rarefaction curves of the number of OTUs at the 97% sequence similarity of two cultivars at different growth stages. (b) Box plots showing the Good’s coverage for the bacterial community in two cultivars. The relative contribution of top ten bacterial phyla (c) and classes (d) in two cultivars at different growth stages. (e) Differentially altered bacterial genera (relative abundance of 0.1% in at least one treatment) in the transgenic rhizosphere as compared to control maize at different growth stages based on student t-test (* denotes *P* < .05; and ** denotes *P* < .01). However, the FDR value for all the genera tested was more than 0.05.

The rarefaction curve of bacterial OTUs at 97% of sequence similarity tended to reach the saturation plateau (Fig. 2a), indicating that large diversity of bacterial community was covered by the present analysis. The Good’s coverage, which reflects the captured microbial diversity in samples, for transgenic and control maize was 81.99% and 82.04%, respectively (Fig. 2b). A total of 50 classified bacterial phyla were detected in all the samples while 2.42% of sequences were unclassified. Proteobacteria, Acidobacteria and Bacteroidetes were the dominant groups, together accounting for 72.13% of the total sequences (Fig. 2c). At the class level, 34 classified groups were found in all the samples whereas 4.44% of total sequences could not be assigned to any class group. Major classes were Gammaproteobacteria, Bacteroidia, Alphaproteobacteria and Deltaproteobacteria, together accounting for 46.14% of total sequences (Fig. 2d).

Taxonomic assignment of identified sequences at the genus level resulted in 558 identified and unidentified groups. Among prominent classified genera (relative abundance >0.1% in at least one treatment), the relative abundances of *Azohydromonas* at stage one, *Mesorhizobium* at stage two, *Bryobacter, Ohtaekwangia* and *Paenisporosarcina* at stage three, *Dongia, Nordella, Paenibacillus* and *Gaiella* at stage four, *Mesorhizobium* at stage five, and *Pedomicrobium* at stage six were higher, whereas that of *Gemmata* and *Segetibacter* at stage two, *Archangium* at stage three, *Cupriavidus* at stage five and *Opitutus* at stage six were lower in transgenic maize rhizosphere than control (*P* < .05) (Fig. 2e). The FDR value obtained from the differential analysis of bacterial community at all taxonomic levels was more than 0.05 (data not shown), indicating that the bacterial composition was not different in transgenic maize and control.

### Difference in Maize Soil Metabolomics Profile of Transgenic and Control Maize

**Figure 3.**
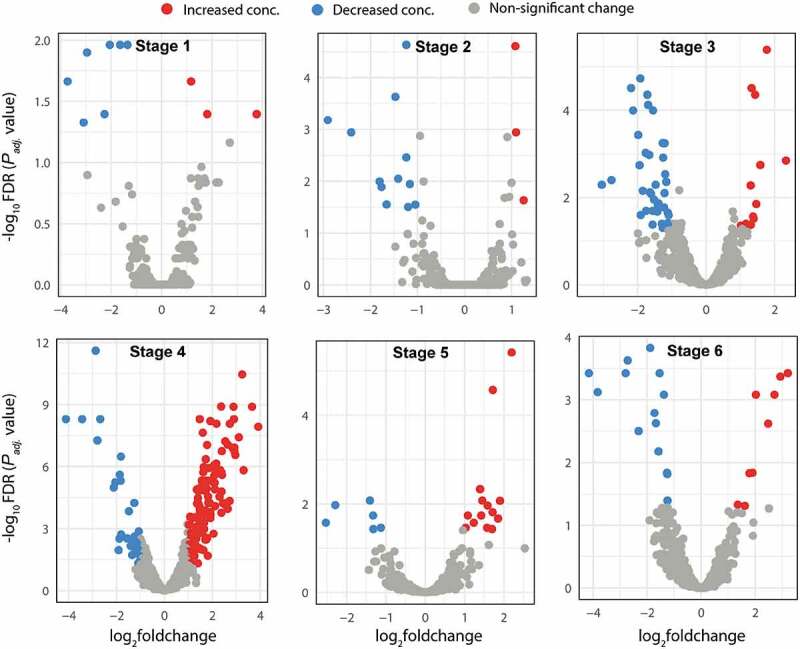
Volcano plot illustrating the differentially altered soil metabolites of transgenic maize as compared to control cultivar at different growth stages. The threshold level for FDR < 0.05 and fold change (2) > 1 or <-1 was set.

Soil metabolomics profiling of two maize cultivars generated a total of 1730 compounds. PLS-DA analysis revealed complete separation between the metabolites of transgenic and control at each growth stage (Supplementary file 2; Fig. S1). Moreover, the degree of change in metabolites increased up to the fourth growth stage and decreased afterward (Fig. 3). In total, 246 metabolites were altered based on threshold of FDR < 0.05; log2foldchange>1 or <-1, with 37 metabolites overlapping at different growth stages (Supplementary file 3). As compared to control, the transgenic maize increased the concentration of 3, 3, 12, 126, 14 and 14 compounds while decreased that of 7, 12, 42, 29, 6 and 9 compounds at stages 1, 2, 3, 4, 5 and 6, respectively (Fig. 3). The results suggest an obvious effect of transgenic maize on soil metabolite profile as compared to control.

The altered metabolites were further assigned to their respective KEGG pathways resulting in a total of 59 KEGG pathways at level III, 43 of those belonging to metabolism at KEGG level 1 (Supplementary file 2; Table S1). The detailed assignment of altered metabolites in both maize cultivars is provided in Table 1. In addition, most of the altered metabolites related to KEGG pathways were observed at growth stage four followed by three.

**Table 1.** The number of differentially altered metabolites belonging to KEGG pathways (level II). The detailed relationship between these metabolites and KEGG pathways are provided in Supplementary file 2; Table S1

	Stage 1		Stage 2		Stage 3		Stage 4		Stage 5		Stage 6	
KEGG pathway level II	Up	Down	Up	Down	Up	Down	Up	Down	Up	Down	Up	Down
Skeleton-based classification	–	–	–	–	–	–	2	–	–	–	–	–
Membrane transport	–	–	–	–	1	2	1	1	–	–	1	–
Signal transduction	–	–	–	–	–	1		–	–	–	–	1
Signaling molecules and interaction	–	–	–	–	–	–	1	–	–	–	–	–
Folding, sorting and degradation	–	–	–	–	1	–	–	–	–	–	–	–
Amino acid metabolism	–	–	1	1	–	1	8	–	–	–	–	1
Biosynthesis of other secondary metabolites	–	–	–	1	1	3	6	–	–	1	–	1
Carbohydrate metabolism	–	1	–	–	–	1	–	–	–	–	–	–
Chemical structure transformation maps	–	–	–	1	–	4	6	–	1	–	–	1
Global and overview maps	–	4	2	3	2	13	37	3	1	–	3	3
Lipid metabolism	–	–	–	1	–	1	3	1	–	–	1	–
Metabolism of cofactors and vitamins	–	–	–	–	–	–	4	2	3	–	1	–
Metabolism of terpenoids and polyketides	–	–	–	–	–	–	–	–	2	–	–	–
Nucleotide metabolism	–	–	–	–	1	1	2	–	–	–	–	–
Nucleotide metabolism	–	–	–	–	–	2	–	–	–	–	–	–
Xenobiotics biodegradation and metabolism	–	–	1	–	–	1	11	–	–	–	–	1
Circulatory system	–	–	–	–	–	1	–	–	–	–	–	–
Digestive system	–	–	–	1	1	–	2	1	–	–	2	–
Endocrine system	–	–	–	–	–	–	1	–	–	–	–	–
Environmental adaptation	–	–	–	–	–	–	2	–	–	–	–	–
Nervous system	–	–	–	–	–	–	1	–	–	–	–	–
Sensory system	–	–	–	–	–	–	1	–	–	–	–	–

### Relationship between Maize Soil Metabolites and Rhizosphere Bacterial Community

**Figure 4.**
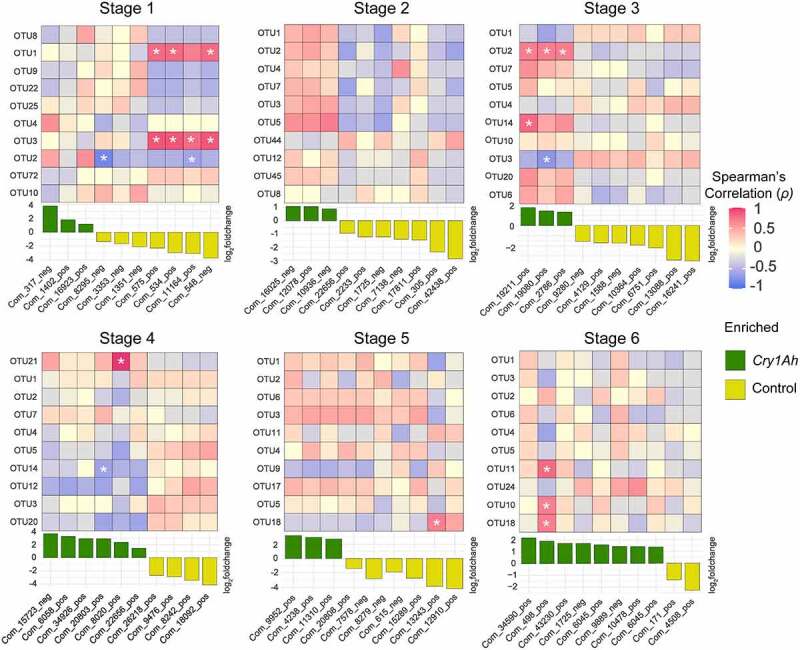
Spearman’s rank correlation analysis between soil metabolites (10 most abundant altered) and rhizosphere bacterial community (10 most abundant OTUs at each stage) of transgenic and control maize cultivars at six different growth stages. The significance of correlations is shown with a white star. The above right panel shows the strength of correlation whereas the downright panel shows if the metabolite was enriched in transgenic or control maize based on FDR value (<0.05) and a fold change (fold_2_change > 1 or < −1).

Since the relative abundances of bacterial OTUs in transgenic and control maize rhizosphere at each growth stage were not different (FDR > 0.5), we only selected the top 10 most abundant OTUs and 10 most abundant altered metabolites at each growth stage, and their relationships were tested using Spearman’s rank correlation coefficient (*ρ*) (threshold, *ρ *> 0.7 or *ρ*< −0.7, and *P* < .05) (Fig. 4).

**Figure 5.**
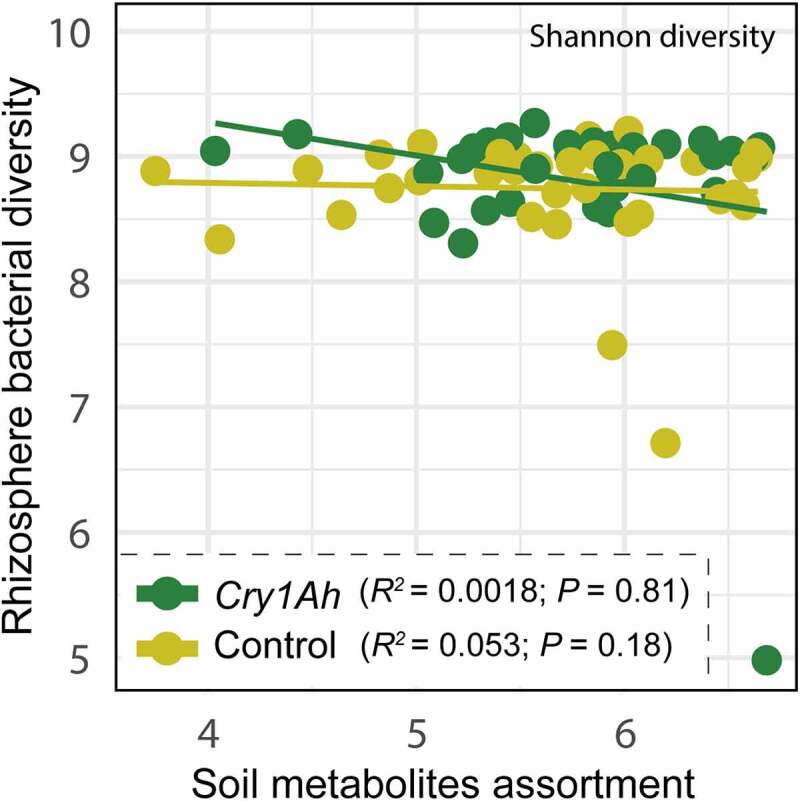
Spearman’s rank correlation analysis between soil metabolites inventory and rhizosphere bacterial community diversity of transgenic and control maize cultivars.

At growth stage one, the relative abundances of OTU1 belonging to family Pyrinomonadaceae, and OTU3 belonging to genus *Stenotrophobacter* were positively correlated with altered metabolites identified as D-gluconic acid, 2-[amino(3-chloroanilino)methylene]malononitrile, Glycyl-L-prolyl-L-lysine (only OTU3) and Prolylleucine, whereas OTU2 belonging to family Nitrosomonadeaceae was negatively correlated with Albiflorin and GPK, and all were decreased in transgenic maize soil. At stage three, OTU2 and OTU14 belonging to the family Nitrosomonadeaceae were positively correlated with the metabolite Rufloxacin, and OTU3 was negatively correlated with 7-Methylguanosine; all were increased in transgenic maize soil. At stage four, OTU21 from order Nostocales was positively correlated to a Triazole compound while OTU14 was negatively correlated with Norfentanyl, both increased in transgenic maize. At stage five, the OTU18 from Gemmatimonadaceae was positively correlated to an unidentified benzodioxin containing compound. The OTU18 along with OTU11 from the family Nitrosomonadeaceae and OUT10 from genus Steroidobacter were positively correlated with Methyl palmitate at stage six which was increased in transgenic maize. Another correlation analysis revealed that the metabolite inventory in transgenic and control maize soils were non significantly (*P* > .05) correlated with the diversity of the bacterial community at all six different growth stages (Fig. 5).

## Discussion

The transfer of transgene flow from GM crops into the soil via crop stubbles or root exudates may cause changes in plant ambient soil environment, potentially impacting the soil biodiversity and ultimately posing a threat to the soil ecosystem.^9^ Therefore, the impact of transgenic crops on soil fauna has been extensively studied in different GM crops, largely resulting in minimal to no effects on biodiversity.^5,24^ In this context, the variable response of soil microbial community to Bt crops has triggered a controversial debate over the last two decades mainly due to the different approaches used to study the rhizosphere microbial community.

Using amplicon sequencing, we found that the alpha and beta diversities of rhizosphere bacterial community of transgenic and control maize did not differ, except the Shannon index at earlier stage which could be the usual transient impact that Bt crops exert on soil microbes.^43,44^ However, obvious differences were found in bacterial beta diversity at different growth stages of both maize cultivars. Likewise, the differences in bacterial relative abundances based on ANOVA were not supported by FDR analysis, which reduces the probability of false-positive in statistical significance level.^45^ These results revealed that the rhizosphere bacterial community varies with growth stages but rather than plant genotypes. Corroborating with these results, several studies have found that GM crops do not pose significant effects on soil microbial community composition and diversity^,30,46^ (Sun et al., ^47^ 2016)^48^ Fan et al.^49^ (2019) found in a 2-year-long trial that insect-resistant transgenic maize carrying *cry1le* gene did not affect the soil fauna. A recent study found that the maize genetically engineered with *mcry1Ab* and *mcray2Ab* genes had minimal to no effect on the rhizosphere bacterial community, and that the different development stages could induce those changes.^30^ When required, a plant can shape and modify its root microbiota through alteration in root exudation.^20,50,51^ The difference in bacterial community structure at different growth stages in our study is certain because of the difference in root exudation chemistry due to variable root biomass and hormonal changes at different stages of growth and development.^52^

Although the rhizosphere bacterial community was not altered in the transgenic maize in our study, we yet analyzed the changes in soil metabolomic profile due to the potential transgene flow.^53^ As predicted earlier,^54^ the metabolomic profile greatly differed between transgenic and non-transgenic maize cultivars at all stages, and the difference was more prominent at mid-stages. It is inevitable because the Bt toxin of GM crops has been shown to impact the root exudation profile.^54–56^ The novel insecticidal protein from Bt crops can be directly released into the soil through root exudation, or their expression can trigger changes in various metabolic pathways resulting in a change in concentration of certain metabolites in the soil surrounding the root system.^57^ Moreover, the prominent difference in soil metabolites during mid-growth stages as compared to other stages could be attributed to the difference in plant physiology. During the transition from vegetative growth to reproductive growth, plants undergo several hormonal and metabolic changes,^58^ which may greatly alter the root metabolism as well as the metabolites diversity in the root ambient soil.

Plant roots exude a proportion of photosynthetically fixed carbon into the soil in the form of different organic compounds, which serve as a major energy reservoir for the soil microbial community.^59^ Therefore, the composition of root-associated microbial community (i.e., in the rhizosphere) is strictly linked with the composition of root exudates of a plant, and any change in root exudation could potentially alter the soil biodiversity in the close vicinity.^60–62^ Despite the obvious differences in metabolic profile in the soils of two maize cultivars at each growth stage, similar to the results of most of the previous studies conducted on cry1Ah insecticidal maize,^34,63,64,65^ no difference in rhizosphere bacterial community was found in our study. Interestingly, the relative abundances of some bacterial OTUs were yet correlated with the altered metabolites in transgenic maize. Even though the different soil metabolic profiles could not alter the bacterial community composition, the association of some bacterial taxa to altered metabolites is not surprising but intuitive.

Based on Spearman’s rank correlation coefficient, we also found that the bacterial diversity was not related to the changes in soil metabolite assortment. Nevertheless, the effects of GM crops on the rhizosphere microbial community are instinct. However, it depends on the spectrum of activity of the transgene protein, the change in root exudation profile and the nature and toxicity of altered metabolites,^43^ Sun et al., 2016;^66,67^ Since examining the nature of all the altered compounds and their level of toxicity on the soil bacterial community was quite troublesome, we screened out the KEGG pathways involving those altered metabolites at different stages. We found that almost third quarter of KEGG pathways with altered metabolites was related to metabolism only, and none of them was found involved in plant–microbe interaction-related pathways such as bacterial chemotaxis,^68^ two-component system,^69^ biofilm formation (Bonlan,^70^ 2001), quorum sensing,^71^ MAPK signaling^72^ and plant hormone signal transduction.^73^ These results suggested that although the metabolite inventory in transgenic maize soil differed from that of non-transgenic maize, the altered metabolic profile was not quite related to the bacterial recruitment process. This may explain why the rhizosphere bacterial community composition, diversity and structure in the *cry1Ah* transgenic maize were not different from that of the non-transgenic maize even with a different soil metabolite composition. However, we strongly recommend investigating the specificity of altered compounds in GM crops and their effects on soil microbial community both in vivo and in vitro to deepen our understandings of the potential effects of transgenic crops on soil biodiversity.

## Conclusion

The environmental biosafety of transgenic crops has always been a big concern since their commercialization. In this study, we assessed the effects of insect-resistant transgenic maize genetically engineered with *cry1Ah* gene on rhizosphere bacterial community and changes in soil metabolites at six different growth stages. We found that the change in rhizosphere bacterial community was related to the plant developmental stages but not to the plant genetic modification, while metabolic profile greatly differed in transgenic and non-transgenic maize. This study revealed that the insect-resistant transgenic maize genetically engineered with *cry1Ah* gene has no obvious effect on the rhizosphere bacterial community. However, the potential role of recognized and unrecognized metabolites altered in transgenic maize soil cannot be overlooked and should be assessed to evaluate the biosafety of GM maize.

## Supplementary Material

Supplemental Material
