# Supplementary material for: Comparison of genetically modified insect-resistant maize and non-transgenic maize revealed changes in soil metabolomes but not in rhizosphere bacterial community
Source: GM Crops Food. 2022 Feb 18;13(1):1–14. doi: 10.1080/21645698.2022.2025725 (PMC8890387; doi:10.1080/21645698.2022.2025725)
Supplement: Supplemental Material [file KGMC_A_2025725_SM1074.zip › Supplementry file 2.docx]

Table S1. The number of differentially altered soil metabolites belonging to KEGG pathways (level I and III). The upward arrow indicates the upregulation whereas the downward arrow indicates the down-regulation of KEGG pathways.

| **KEGG Level I** | **KEGG Level III** | **Stage 1** | | **Stage 2** | | **Stage 3** | | **Stage 4** | | **Stage 5** | | **Stage 6** | |
| --- | --- | --- | --- | --- | --- | --- | --- | --- | --- | --- | --- | --- | --- |
|  |  |  |  |  |  |  |  |  |  |  |  |  |  |
| Drug Development | Naphthalene family |  |  |  |  |  |  | 2 |  |  |  |  |  |
| Environmental Information Processing | ABC transporters |  |  |  |  | 1 | 2 | 1 | 1 |  |  | 1 |  |
| Environmental Information Processing | Plant hormone signal transduction |  |  |  |  |  | 1 |  |  |  |  |  |  |
| Environmental Information Processing | AMPK signaling pathway |  |  |  |  |  |  |  |  |  |  |  | 1 |
| Environmental Information Processing | Neuroactive ligand-receptor interaction |  |  |  |  |  |  | 1 |  |  |  |  |  |
| Genetic Information Processing | Sulfur relay system |  |  |  |  | 1 |  |  |  |  |  |  |  |
| Human Diseases | Chemical carcinogenesis - receptor activation |  |  |  |  |  |  | 1 |  |  |  |  |  |
| Human Diseases | Diabetic cardiomyopathy |  |  |  |  |  |  | 1 |  |  |  |  |  |
| Human Diseases | Nicotine addiction |  |  |  |  |  |  | 1 |  |  |  |  |  |
| Metabolism | Cysteine and methionine metabolism |  |  |  |  |  |  | 1 |  |  |  |  |  |
| Metabolism | Phenylalanine metabolism |  |  | 1 |  |  | 1 | 2 |  |  |  |  | 1 |
| Metabolism | Tryptophan metabolism |  |  |  | 1 |  |  | 3 |  |  |  |  |  |
| Metabolism | Tyrosine metabolism |  |  |  |  |  |  | 2 |  |  |  |  |  |
| Metabolism | Indole alkaloid biosynthesis |  |  |  |  |  |  | 1 |  |  |  |  |  |
| Metabolism | Isoflavonoid biosynthesis |  |  |  |  |  |  | 1 |  |  |  |  |  |
| Metabolism | Phenylpropanoid biosynthesis |  |  |  | 1 |  |  | 2 |  |  |  |  |  |
| Metabolism | Tropane, piperidine and pyridine alkaloid biosynthesis |  |  |  |  |  | 2 |  |  |  |  |  |  |
| Metabolism | Flavonoid biosynthesis |  |  |  |  | 1 | 2 |  |  |  | 1 |  |  |
| Metabolism | Caffeine metabolism |  |  |  |  |  | 1 |  |  |  |  |  |  |
| Metabolism | Isoquinoline alkaloid biosynthesis |  |  |  |  |  |  |  |  |  |  |  | 1 |
| Metabolism | Pentose phosphate pathway |  | 1 |  |  |  | 1 |  |  |  |  |  |  |
| Metabolism | Biosynthesis of alkaloids derived from ornithine, lysine and nicotinic acid |  |  |  |  |  |  | 1 |  |  |  |  |  |
| Metabolism | Biosynthesis of alkaloids derived from shikimate pathway |  |  |  |  |  | 1 |  |  |  |  | 1 |  |
| Metabolism | Biosynthesis of phenylpropanoids |  |  |  | 1 |  |  | 3 |  |  |  |  |  |
| Metabolism | Biosynthesis of plant secondary metabolites |  |  |  |  |  | 2 | 1 |  | 1 |  |  |  |
| Metabolism | Biosynthesis of alkaloids derived from histidine and purine |  |  |  |  |  | 1 |  |  |  |  |  |  |
| Metabolism | Biosynthesis of plant hormones |  |  |  |  |  | 1 |  |  |  |  |  |  |
| Metabolism | Biosynthesis of amino acids |  |  |  |  |  |  | 1 |  |  |  |  |  |
| Metabolism | Biosynthesis of cofactors |  |  |  | 1 |  |  | 5 | 1 |  |  | 1 |  |
| Metabolism | Biosynthesis of secondary metabolites |  | 1 |  | 1 |  | 3 | 4 | 1 |  |  | 1 | 1 |
| Metabolism | Degradation of aromatic compounds |  |  |  |  |  |  | 1 |  |  |  |  |  |
| Metabolism | Metabolic pathways |  | 1 | 1 | 1 | 2 | 6 | 18 | 1 | 1 |  | 1 | 1 |
| Metabolism | Microbial metabolism in diverse environments |  | 1 | 1 |  |  | 3 | 8 |  |  |  |  | 1 |
| Metabolism | Carbon metabolism |  | 1 |  |  |  | 1 |  |  |  |  |  |  |
| Metabolism | Arachidonic acid metabolism |  |  |  |  |  |  | 1 |  |  |  |  |  |
| Metabolism | Steroid hormone biosynthesis |  |  |  |  |  |  | 2 | 1 |  |  |  |  |
| Metabolism | alpha-Linolenic acid metabolism |  |  |  |  |  | 1 |  |  |  |  |  |  |
| Metabolism | Secondary bile acid biosynthesis |  |  |  | 1 |  |  |  |  |  |  | 1 |  |
| Metabolism | Biotin metabolism |  |  |  |  |  |  | 1 |  |  |  |  |  |
| Metabolism | Nicotinate and nicotinamide metabolism |  |  |  |  |  |  | 1 | 1 | 1 |  |  |  |
| Metabolism | Porphyrin and chlorophyll metabolism |  |  |  |  |  |  | 1 |  |  |  |  |  |
| Metabolism | Riboflavin metabolism |  |  |  |  |  |  | 1 | 1 |  |  | 1 |  |
| Metabolism | Tetracycline biosynthesis |  |  |  |  |  |  |  |  | 1 |  |  |  |
| Metabolism | Biosynthesis of type II polyketide products |  |  |  |  |  |  |  |  | 1 |  |  |  |
| Metabolism | Pyrimidine metabolism |  |  |  |  | 1 | 1 | 2 |  |  |  |  |  |
| Metabolism | Purine metabolism |  |  |  |  |  | 2 |  |  |  |  |  |  |
| Metabolism | Aminobenzoate degradation |  |  |  |  |  |  | 3 |  |  |  |  |  |
| Metabolism | Bisphenol degradation |  |  |  |  |  |  | 1 |  |  |  |  |  |
| Metabolism | Caprolactam degradation |  |  |  |  |  |  | 1 |  |  |  |  |  |
| Metabolism | Metabolism of xenobiotics by cytochrome P450 |  |  |  |  |  |  | 3 |  |  |  |  |  |
| Metabolism | Styrene degradation |  |  | 1 |  |  | 1 | 2 |  |  |  |  | 1 |
| Metabolism | Toluene degradation |  |  |  |  |  |  | 1 |  |  |  |  |  |
| Organismal systems | Adrenergic signaling in cardiomyocytes |  |  |  |  |  | 1 |  |  |  |  |  |  |
| Organismal systems | Bile secretion |  |  |  | 1 |  |  | 2 |  |  |  | 1 |  |
| Organismal systems | Vitamin digestion and absorption |  |  |  |  | 1 |  |  | 1 |  |  | 1 |  |
| Organismal systems | Thyroid hormone synthesis |  |  |  |  |  |  | 1 |  |  |  |  |  |
| Organismal systems | Thermogenesis |  |  |  |  |  |  | 2 |  |  |  |  |  |
| Organismal systems | Serotonergic synapse |  |  |  |  |  |  | 1 |  |  |  |  |  |
| Organismal systems | Inflammatory mediator regulation of TRP channels |  |  |  |  |  |  | 1 |  |  |  |  |  |


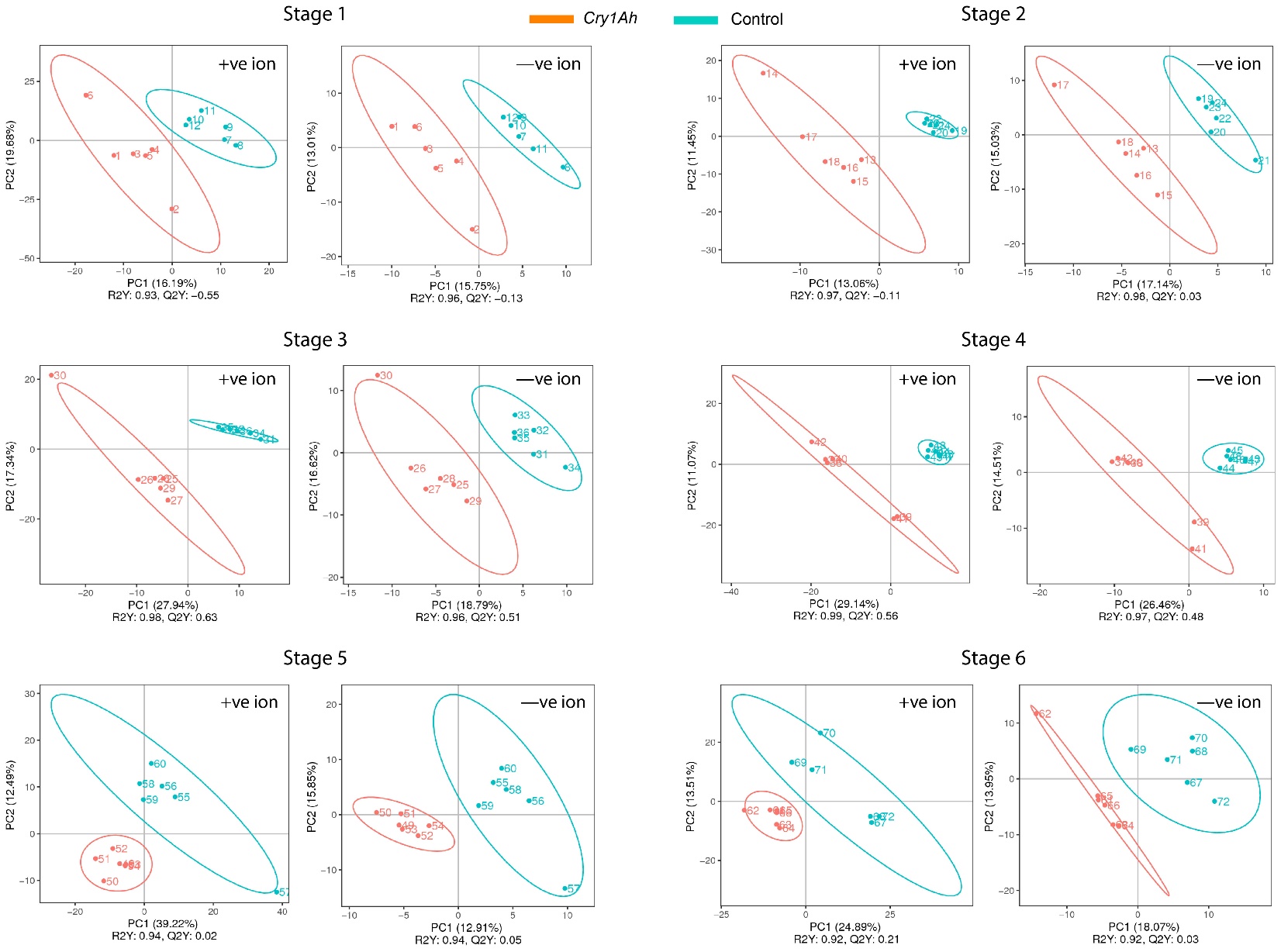
Figure S1. PLS-DA analysis showing the difference in metabolomic profile between transgenic and non-transgenic maize soils at different growth stages. The analysis was conducted separately on metabolites with positive and negative ions at each stage.
